# Supplementary material for: Chronic Diseases and Employment: Which Interventions Support the Maintenance of Work and Return to Work among Workers with Chronic Illnesses? A Systematic Review
Source: Int J Environ Res Public Health. 2019 May 27;16(10):1864. doi: 10.3390/ijerph16101864 (PMC6572561; doi:10.3390/ijerph16101864)
Supplement: Supplementary file 1 [file ijerph-16-01864-s001.pdf]

**Table S1.** Study characteristics: methodological characteristics, outcome measures and results.

| Nr. | First author (year), country | Chronic disease | Intervention group | | Control group | | Follow-up after intervention | Primary outcome | Secondary outcome | Effect (RR, OR, HR, CI, mean SD, median) |
|---|---|---|---|---|---|---|---|---|---|---|
| . | | | **N=<br>female n=<br>male n=** | **Socio-demographic data age, education, employment** | **N=<br>female: n=<br>male: n=** | **Socio-demographic data age, education, employment** | | | | |
| 1 | Bakker et al. (2007), Netherlands | Stress-related mental disorders | N=227<br>female: n=153(67%)<br>male: n=74(33%) | mean age (y): 41.97<br>Level of education:<br>low 59(27%)<br>intermediate 102(50%)<br>high 57(28%)<br>Employment: -- | N=206<br>female: n=134(65%)<br>male: n=72(35%) | mean age (y): 39.5<br>Level of education:<br>low 59(27%)<br>intermediate 102(50%)<br>high 57(28%)<br>Employment: -- | 12 months | sick leave in days until full return to work (RTW) | self-reported symptomps of distress, depression, anxiety, somatisation | HR=1.06; 95% CI 0.87-1.29 |
| 2 | Bendix et al. (2000), Denmark | Chronic low back pain (CLBP) | N=48<br>The female-to-male ratio was 68 to 31 for both group | median age (y): 42<br>Work capable (n=; Median(%)):<br>n= 59; 31(53%) | N=51<br>The female-to-male ratio was 68 to 31 for both group | median age (y): 42<br>Work capable (n=; Median(%)):<br>n= 68; 27(40%) | 1 year | work capable | | 58% vs 42%, p=0.09 |
| 3 | Bergström et al. (2012), Sweden | Chronic neck pain (NP) and/or low back pain (LBP) | AC: N=62<br>female: n=31<br>ID: N=52<br>female: n=31<br>DYS: N=80<br>female: n=44 | AC: mean age: 43,8;<br>Education: school n=31, high school n=22, post high school n=8<br>Employment<br>Gainfully employed 54 (87.1)<br>ID: mean age: 41,8;<br>Education: school n=28, high school n=20, post high school n=4<br>Employment<br>Gainfully employed 45 (86.5)<br>DYS: mean age: 43,8;<br>Education: school n=31, high school n=22, post high school n=8<br>Employment<br>Gainfully employed 63 (79.7) | | AC: N=18<br>ID: N=11<br>DYS: N=17 | 4 × 4 repeated measure with a 10-year follow-up | registered sickness absence (including disability pension) after rehabilitation during a 10-year follow-up period | | point estimate differences<br>BM: p = 0.236 (–17.77, CI:−39.5 to 9.9),<br>PT: p = 0.373 (11.07, CI:−13.6 to 35.8),<br>CBT: p = 0.592 (–6.09, CI:−28.7 to 16.5)<br>and adjusted: BM: p = 0.146 (–16.08, CI:−38.0 to 5.8),<br>PT: p = 0.960 (–0.55, CI:−22.5 to 21.4),<br>CBT: p = 0.416 (–7.79, CI:−26.9 to 11.3) |

| Nr. | First author (year), country | Chronic disease | Intervention group | | Control group | | Follow-up after intervention | Primary outcome | Secondary outcome | Effect (RR, OR, HR, CI, mean SD, median) |
|---|---|---|---|---|---|---|---|---|---|---|
| . | | | **N= female n= male n=** | **Socio-demographic data age, education, employment** | **N= female: n= male: n=** | **Socio-demographic data age, education, employment** | | | | |
| 4 | Christensen et al. (2003), Denmark | Different musculoskeletal disorders | Video group (VG), cafe group (CG), training group (TG) Altogether, 90 patients were included: 60 females and 30 males a mean age of 45 (range: 24–60) | Employment: 38% of the patients were working or were full-time students, 37% were on sick leave, 18% retired, 3% in rehabilitation program, and 4% unemployed | | | 1 and 2 years | Work ability | | . At the 1-year follow-up, significantly more patients in VG and TG were on sick leave compared with CG (P<0.02), and significantly fewer patients were working in VG compared with the two other groups (P < 0.04). Significantly fewer patients had applied for pension in CG and TG compared with VG (P < 0.03). At 2-year follow-up, only 8% of patients in CG were on sick leave compared with VG (35%) and TG (32%) (P < 0.05). At the 2-year follow-up, 52% in VG, 22% in CG, and 64% in TG were fired because of low back pain (P < 0.01). |
| 5 | Dalgaard et al. (2017), Denmark | Adjustment disorder or reactions to severe stress (ICD 10 code: F43.2–F43.9), or mild depressive episode (F32.0) | N=58 female n(%)=43(74.1), male n(%)=15(25.9) | Mean age: 45 (range 28–60) Basic education 9th grade or less 8(13.8), 10–12 years 49(84.5), Higher education (years) short (<3) 21(36.2), medium (3–4) 28(48.3), long (>4) 9(15.5) Employment: on sick leave (full or part time) | A group (which received a clinical assessment) N=56 B group (no assessment) N=49 A: female n(%)=40(71.4), male n(%)=16(28.6) B: female n(%)=37(75.5), | Mean age: group A = 44 (range 29–63); group B = 46 (range 26–62) Basic education 9th grade or less A group 5(8.9), B group 3(6.1) 10–12 years A group 50(89.3), B group 39(79.6) Higher education (years) short (<3) A group 27(48.2), B group 26(53.1) medium (3–4) | 16 and 44 weeks | | Lasting RTW | Adjusted model (age +gender +weeks+diagnosis) HR (95% CI, p-value) at 16 weeks follow-up. Intervention group HR= 1.57 (0.87–2.82, p= 0.13), A group HR=1 (--), B group HR=0.66 (0.31–1.42, p= 0.29) Adjusted model (age +gender +weeks+diagnosis) HR(95% CI, p-value) at 44 weeks follow-up Intervention group 1.50( 0.85–2.62, p=0.09), A group 1(-), B group 0.60(0.36–1.00, p=0.05) Median and mean numbers of weeks (95% CI) at 44 weeks |

| Nr. | First author (year), country | Chronic disease | Intervention group | | Control group | | Follow-up after intervention | Primary outcome | Secondary outcome | Effect (RR, OR, HR, CI, mean SD, median) |
|---|---|---|---|---|---|---|---|---|---|---|
| . | | | **N= female n= male n=** | **Socio-demographic data age, education, employment** | **N= female: n= male: n=** | **Socio-demographic data age, education, employment** | | | | |
| | | | | | male n(%)=12(24.5) | A group 24(42.9), B group 15(30.6) long (>4) A group 5(8.9), B group 1(2.0) Employment: on sick leave (full or part time) | | | | follow-up Median Intervention group 15(12–19), A group 19(15–30), B group 32( 22– -) |
| 6 | De Buck et al. (2005), Netherlands | Chronic rheumatic disease | N=74 female: n=41(55), male: n=33(45) | Age, median (range) years: 43 (21–57) Education level: High 15 (20) Medium 37 (50) Low 22 (30) | N=66 female: n=38(58), male: n=28(42) | Age, median (range) years: 44 (24–58) Education level: High 10 (15) Medium 39 (59) Low 17 (26) | at baseline, 6 months, 12 months, 18 months, 24 months | job loss (complete work disability or unemploy ment) | satisfaction with the job, pain, fatigue, physical functioning, and quality of life | no statistically significant difference in the proportion of patients with permanent job loss between the groups at any time point p=0.13 |
| 7 | Detaille et al. (2013), Netherlands | Neoplasms, endocrine, nutritional and metabolic diseases, diseases of: respiratory system, digestive system, musculoskele tal system and connective tissue, other diseases | N=44 Women (%) n=70 | Age (years mean, SD) 46.6, 8.0 Education (%) Lower 20 Middle 25 High 55 | N=35 Women (%) n=66 | Age (years mean, SD) 48.9, 11.4 Education (%) Lower 38 Middle 27 High 35 | Intervention group at baseline, 6 weeks and 8 months control group at baseline and at 8 months | self-efficacy at work and attitude towards self-manageme nt at work (importanc e and enjoyment) | job satisfaction and intention to change job | **self-efficacy**: intervention group (estimated marginal means) = 15.4, SE=3.0, CI 9.4–21.4; control group (estimated marginal means) = 15.2, SE=2,9, CI 9.4–21.0, p value = 0.956 **attitude**: intervention group (estimated marginal means) = 17.4, SE=2.5, CI 12.5–22.3; control group (estimated marginal means) = 10.3, SE=2.4, CI 5.4–15.1, p value = 0.030** |

| Nr. | First author (year), country | Chronic disease | Intervention group | | Control group | | Follow-up after intervention | Primary outcome | Secondary outcome | Effect (RR, OR, HR, CI, mean SD, median) |
|---|---|---|---|---|---|---|---|---|---|---|
| . | | | **N=<br>female n=<br>male n=** | **Socio-demographic data age, education, employment** | **N=<br>female: n=<br>male: n=** | **Socio-demographic data age, education, employment** | | | | |
| 8 | Friedrich et al. (2005), Austria | Chronic and recurrent low back pain (LBP) | N=44 at baseline, N=26 5 years follow-up female: n=13(23.2), male: 13(23.2) | Age (y): 47.5, SD 10.6 | N=49 at baseline, N=30 5 years follow-up female: n=18(32.1), male: n=12(21.4) | Age (y): 51.4, SD 10.9 | 3.5 weeks, 4 months, 12 months and 5 years | disability scores, pain intensity, working ability | body mass index, the number of LBP episodes requiring treatment, and the use of health care | pain intensity (control group SD=32.59; motivational group SD=17.29), in the low back outcome scale (control group SD=17.07; motivational group SD=10.67) and working ability (control group SD=3.41; motivational group SD=1.47) |
| 9 | Lambeek et al. (2010), Netherlands | CLBP | N=66<br>female n(%): 29 (44)<br>male n(%): 37 (56) | Mean (SD) age (years): 45.5 (8.9) Level of education: Low 14 (21) Intermediate 34 (52) High 18 (27) | N=68<br>female n(%): 27 (40) male n(%): 41 (60) | Mean (SD) age (years): 46.8 (9.2) Level of education: Low 23 (34) Intermediate 32 (47) High 13 (19) | 12 months | sick leave | improvements in functional status and pain intensity | HR= 1.90 (95% CI 1.18 - 2.76, P=0.004). During the 12 months of follow-up the median number of days of sick leave (including recurrences) in the integrated care group was 82 (interquartile range 51 to 164 days) compared with 175 (91 to 365) in the usual care group. This difference was significant (Mann-Whitney U test; P=0.003) |
| 10 | Linton et al. (2000), Sweden | Spinal pain | Cognitive-Behavior Therapy group (CBT)<br>N=107<br>female (%) 70 | Age mean (y) 44<br>Education (%): compulsory 19, technical high school 31, high school 13, university 37<br>Employment: employed 79, unemployed 07, student 08, other 06 | Pamphlet group (PG)<br>N=70<br>female (%) 71<br>Information Package group (IG)<br>N=66<br>female (%) 74 | PG**:** age mean (y) 45<br>Education (%): compulsory 19, technical high school 35, high school 16, university 30<br>Employment: employed 69, unemployed 13, student 10, other 08<br>IG: age mean (y) 44, Education (%): compulsory 17, technical high school 32, high school 16, university 35 | 12 months | long-term sick leave, health care use, risk for disability | | Days of sick leave Point Estimate (95% CI): PG: before 3.0(20.5–6.4) follow-up 13.0(1.2–24.8) IG: before 5.0(0–10.0), follow-up 19.4(3.4–35.3) CBT: before 3.0(0.5–5.5), follow-up 2.6(-1.6–6.7)- Risk for long-term disability: RR=9.3(1.2–70.8) |

| Nr. | First author (year), country | Chronic disease | Intervention group | | Control group | | Follow-up after intervention | Primary outcome | Secondary outcome | Effect (RR, OR, HR, CI, mean SD, median) |
|---|---|---|---|---|---|---|---|---|---|---|
| . | | | **N= female n= male n=** | **Socio-demographic data age, education, employment** | **N= female: n= male: n=** | **Socio-demographic data age, education, employment** | | | | |
| | | | | | | Employment: employed 73, unemployed 10, student 08, other 09 | | | | |
| 11 | McGonagle et al. (2014), USA | Ankylosing spondylitis, nerve injury, neuropathy, fibromyalgia, diabetes 1+2, multiple sclerosis), psoriatic arthritis), psychiatric illness and Sjögren's syndrome | Coaching group (CoG) N=30 | Age mean(SD): n=38.30 (8.2) Participants were predominately female (86%), were generally well educated (73% had a 4-year college degree or graduate degree) | Waitlisted control (WC) N=29 | Age mean(SD): n=39.07 (7.79) Participants were predominately female (86%), were generally well educated (73% had a 4-year college degree or graduate degree) | 12 weeks | Job self-efficacy, work ability, exhaustion burnout and other | | CoG: Job self-efficacy: Pre mean(SD) 3.42 (.66), Post mean(SD) 3.70 (.58) work ability Pre 3.39 (.75), Post 3.82 (.39) exhaustion burnout Pre 2.92 (.40), Post 2.65 (.46)<br>WG: Job self-efficacy: Pre mean(SD) 3.62 (.57), Post mean(SD) 3.70 (.58) work ability Pre 3.36 (.66), Post 3.82 (.39) exhaustion burnout Pre 2.86 (.38),<br>Post 2.89 (.34) |
| 12 | Nieuwenhuijsen et al. (2017), Netherlands | Work-related chronic stress complaints | Intervention group (IG)N=28 Gender (% women) n=73 | Age years mean (SD): n=43 (8.0)<br>Employment: Work-hours RTW at baseline (% of contract hours) mean(SD) 7.3 (16.70 | Placebo group (PG) N=28, Control group (CG) N=28 PG: Gender (% women) n=69<br>CG: Gender (% women) n=66 | PG: Age years mean (SD): n=47 (9.7)<br>Employment: Work-hours RTW at baseline (% of contract hours) mean(SD) 13.2 (20.44)<br>CG: Age years mean (SD): n=40 (8.9)<br>Employment: Work-hours RTW at baseline (% of contract hours) mean (SD) 6.9 (17.80) | at baseline, and after 6, 12 and 24 weeks | RTW percentage | Fatigue, stress, quality of life | % work-hours RTW (0–100) after 24 weeks<br>IG: 94.7(80.63), PG: 88.2(58.54), CG: 62.5(72.31) |
| 13 | Ntsiea et al. (2017), South Africa | Stroke | N=40<br>female n(%): n=19 (48),<br>male n(%): n=21 (52) | Average age (years mean, SD): 45 (8.5)<br>Educational level n (%)<br>Degree: 6 (15)<br>Grade 12 + 3 years: 7 (17) | N=40<br>female n(%): n=20 (50), male n(%): n=20 (50) | Average age (years mean, SD): 44 (8.9)<br>Educational level n (%)<br>Degree: 0 (0)<br>Grade 12 + 3 years: 9 (22) | 3 months, 6 months | RTW rate | activities of daily living (ADLs), mobility, basic | 3 months follow-up<br>Returned to work n(%):<br>Intervention group n=11 (27), control group n= 5 (12)<br>6 months follw-up |

| Nr. | First author (year), country | Chronic disease | Intervention group | | Control group | | Follow-up after intervention | Primary outcome | Secondary outcome | Effect (RR, OR, HR, CI, mean SD, median) |
|---|---|---|---|---|---|---|---|---|---|---|
| . | | | **N= female n= male n=** | **Socio-demographic data age, education, employment** | **N= female: n= male: n=** | **Socio-demographic data age, education, employment** | | | | |
| | | | | Grade 12 or equivalent: 15 (38)<br>Grade 11: 10(25)<br>Grade 7 and below: 2 (5) | | Grade 12 or equivalent: 9 (22) Grade 11: 17 (43)<br>Grade 7 and below: 5 (13) | | | cognitive function and perceived quality of life | Returned to work n(%): intervention group n=24(60), control group n=8(20); OR=5.2, 95% CI (1.8 – 15.0) |
| 14 | Varekamp et al. (2011), Netherlands | Diseases of: musculoskeletal system and connective tissue, nervous system, digestive system, endocrine, nutritional and metabolic diseases, neoplasms, e respiratory system, circulatory system | N=64<br>female n(%): n=53(83)<br>male n(%): n=11(17) | Mean age (y): 46.1<br>Education:<br>lower 3%<br>middle 36%<br>higher 64%<br>Employment:<br>Work capacity, physical: (very) bad 13, moderate 55, (very) good 33<br>Work capacity, mental: (very) bad 14, moderate 47, (very) good 39 | N=58<br>female n(%): n=38(66%)<br>male n(%): n=20(34%) | Mean age (y): 46.1<br>Education:<br>lower 3%<br>middle 36%<br>higher 64%<br>Work capacity, physical: (very) bad 24 moderate 47 (very) good 29<br>Work capacity, mental: (very) bad 19, moderate 40, (very) good 41 | 4, 8, 12 and 24 months | 1. self-efficacy in solving work- and disease-related problems, 2. fatigue, and 3. job dissatisfaction. | 1. job maintenance itself,<br>2. work-related problems,<br>3. quality of life,<br>4. acquired work accommodations,<br>5. burnout, and<br>6. worries about work. | Time effect F=28.7, p=0.000.<br>Time × interaction effect F=5.6, p=0.000.<br>Job maintenance OR=1.42 , 95% CI=(0.44–4.64) |
| 15 | Wong et al. (2008), Hong Kong | mental illness | N=46<br>female: n=30<br>male: n= 25 | Age (M±SD) 32.4±8.9<br>Employment: Months working at a paid job in the past 5 years (M±SD) 18.4±17.3 | N=46<br>female: n=16<br>male: n= 21 | Age (M±SD) 34.7±9.4<br>Employment: Months working at a paid job in the past 5 years (M±SD) 13.0±15.6 | 6, 12, 18 months | Vocational: competitive employment rates, time to first job, total days employed, and total earnings | Non-vocational: psychiatric symptoms and self-perceived quality of life | 70% versus 29%; OR=5.63, CI=2.28–13.84 |

**Table S2.** Characteristics of intervention programs.

| Nr. | First author (year), country | Chronic disease | Objectives of intervention | Intervention methods | Structure of intervention | Number and discipline of trainer or counsellors | Recruitment procedures |
|---|---|---|---|---|---|---|---|
| 1 | Bakker et al. (2007), Netherlands | Stress-related mental disorders | Assessment of effectiveness of Minimal intervention for stress-related mental disorders with sick leaves (MISS) in primary care, which is intended to reduce sick leave and prevent chronicity of SMD symptoms in patients | Primary care physicians (PCPs) were randomized to an intervention group that was trained to deliver a minimal intervention for stress-related mental disorders, or to a control group that delivered care as usual. Distressed patients on sick leave visiting the practices of both PCP groups were screened, included, and followed up for one year | Over a period of 6–10 week, the PCPs randomized into the intervention group received training in the MISS. The training comprised two sessions of 3.5 h and two regular follow-up sessions of 2 h (total 11 h). | Primary care physicians and an occupational physician | From network of Department of General Practice of the VU University Medical Center a total of 46 PCPs signed informed consent, both for participating in trial and for being randomized to either the intervention training or to the usual care (UC) group. Between September 2003 and October 2004, a screening letter was sent to the source population of 22,740 patients. The overall response percentage on our screening method was 51.5%; this was measured in a group of 336 randomly selected attenders. A total of 433 patients (1.9% of 22,470) were included in the study, 66.3% of whom were women |
| | Bendix et al. (2000), Denmark2 | Chronic low back pain (CLBP) | To compare the effect of a comprehensive functional restoration program involving intensive physical training, ergonomic training and behavioral support with that of an outpatient physical training program | Functional Restoration (FR) Program: 8:00–9:00 Aerobic exercises 9:00–10:00 Fitness machines 10:00–11:30 Occupational therapy (work-hardening therapy) 11:30–12:00 Lunch 12:00–1:30 Group therapy (psychology) 1:30–2:00 Stretching exercises 2:00–3:00 Theory: basic anatomy, pathology including radiographs, sports medicine, etc. 3:00–4:00 Recreational activities, follow-up treatment. Outpatient Intensive Physical Training (OIT) | FR: 3 weeks, 8 hours daily OIT: 1.5 hour three times per week for 8 weeks. | Physical therapist, occupational therapist, psychologist | The prospective participants were referred from general practitioners, specialists, other hospital departments, or insurance companies |

| Nr. | **First author (year), country** | **Chronic disease** | **Objectives of intervention** | **Intervention methods** | **Structure of intervention** | **Number and discipline of trainer or counsellors** | **Recruitment procedures** |
|---|---|---|---|---|---|---|---|
| 3 | Bergström et al. (2012), Sweden | Chronic neck pain (NP) and/or low back pain (LBP) | Interaction between treatment content and subgroups according to the Swedish version of the Multidimensional Pain Inventory (MPI-S) on the effect on sickness absence during a 10-year follow-up | The interventions consisted of Behavioral-oriented Physiotherapy (PT), Cognitive Behavioral Therapy (CBT), Behavioral Medicine Rehabilitation (BM), and a "treatment-as-usual" control group (CG).<br>PT included individual goal setting, gradually increased exercises to improve muscular endurance, aerobic training, pool training, relaxation techniques and body awareness therapy. Homework assignments for physical activities were given according to the individual's interests and problem areas.<br>CBT intervention was to improve the subjects' ability to manage pain and to resume a normal level of activity. BM was a multidisciplinary program, in which all parts of the PT and CBT programs were included. BM was given on a full-time basis (40 scheduled hours per week). | PT: Each participant was assigned to an individually tailored training program, which was carried out on a part-time basis (approximately 20 scheduled hours per week).<br>CBT: individuals were required to participate in scheduled activities for approximately 13–14 hours per week. BM was given on a full-time basis (40 scheduled hours per week).<br>Multidisciplinary programs, in which all parts of the PT and CBT programs were included. BM was given on a full-time basis (40 scheduled hours per week) | Rehabilitation clinics in four large cities in Sweden, licensed physiotherapists, psychologists and physicians | The subjects were recruited from the AFA health insurance registry. Inclusion criteria included continuous sickness absence for 1–6 months due to non-specific spinal pain, 18–60 years of age and fluency in Swedish. Participants were recruited consecutively and rehabilitation took place from May 1995 to March 1998. The follow-up was completed in March 2008. Three different patient groups have empirically been derived from the MPI: interpersonally distressed (ID) patients who are characterized by low levels of social support, dysfunctional (DYS) patients who are characterized by high pain severity, disability and affective distress, and adaptive copers (AC) who report a more successful adjustment to chronic pain |

| Nr. | First author (year), country | Chronic disease | Objectives of intervention | Intervention methods | Structure of intervention | Number and discipline of trainer or counsellors | Recruitment procedures |
|---|---|---|---|---|---|---|---|
| 4 | Christensen et al. (2003), Denmark | Different musculoskeletal disorders | Effect of three different rehabilitation strategies for lumbar spinal fusion patients | Video group participants watched a video of exercises for training and were subsequently and only once provided instruction regarding their use. The back-cafe´ group was provided the same program as the video group, but as a supplement met with a back-cafe´ with other fusion-operated patients three times over an 8-week period. The training group was provided physical therapy training twice weekly for 8 weeks | 8 weeks | Physical therapist | From November 15, 1996 to September 20, 1999, 115 patients, each of whom had undergone a lumbar spondylodesis operation 3 months previously, were invited to participate in the present randomized, prospective investigation. They all had severe chronic low back pain caused by localized lumbar or lumbosacral segmental instability caused by isthmic spondylolisthesis grades I or II, primary degeneration (no previous surgery), secondary degeneration after decompressive surgery, or accelerating degeneration after decompressive surgery |
| 5 | Dalgaard et al. (2017), Denmark | Adjustment disorder or reactions to severe stress (ICD 10 code: F43.2–F43.9), or mild depressive episode (F32.0) | The efficacy of work-focused cognitive behavior therapy (CBT) combined with an optional workplace intervention for patients on sick leave due to work-related stress complaints | (i) identifying work-related stressors, (ii) modifying cognitive and behavioral coping strategies, (iii) providing psycho-education about work related stress, (iv) assigning homework, and (v) assisting participants in planning RTW | The intervention consisted of six, one-hour sessions with individual work-focused CBT conducted by a psychologist over 16 weeks | Psychologist | By the True Random Number Generator (www.random.org) and randomized to either the clinical assessment or control group B. Second, participants included on the basis of the clinical assessment were randomized to either the intervention or control group A. |

| Nr. | **First author (year), country** | **Chronic disease** | **Objectives of intervention** | **Intervention methods** | **Structure of intervention** | **Number and discipline of trainer or counsellors** | **Recruitment procedures** |
|---|---|---|---|---|---|---|---|
| 6 | De Buck et al. (2005), Netherlands | Chronic rheumatic disease (diagnosis of RA, AS, psoriatic arthritis, reactive arthritis, SLE, or scleroderma) | Effectiveness of a multidisciplinary job-retention vocational rehabilitation (VR) program in patients who were at risk for job loss | Systematic assessment followed by education, vocational counseling, guidance, and medical or nonmedical treatment | The total duration of the intervention varied, and lasted on average between 4 and 12 weeks | Rheumatologist, occupational physician, coordinator | Between March 1999 and June 2001, patients were recruited at the outpatient rheumatology departments of Leiden University Medical Center and 10 nonacademic hospitals within the region of Leiden, the Netherlands. Participants were between 18 and 63 years of age and had a chronic rheumatic disease. All patients had a paid job (working full time or part time or on sick leave, either with or without a partial disability pension) and were having a self-perceived, disease-related problem at work that threatened their ability to work. |
| 7 | Detaille et al. (2013), Netherlands | Neoplasms, endocrine, nutritional and metabolic diseases, diseases of: respiratory system, digestive system, musculoskeletal system and connective tissue, other diseases | Self-Management Program for workers with a chronic disease as an adaptation of the Chronic Disease Self-Management Program of Stanford University (CDSMP). The aim of training was to influence the determinants of self-management at work and finally to improve self-management behavior at work. | The original CDSMP focuses on personal lifestyle factors and disease-related factors like coping with symptoms of the disease. In the adapted program included work-related factors like, for instance, how to communicate with colleagues and supervisors, acquiring resources at work and dealing with symptoms of the disease at work. | The course consists of six weekly sessions, each two and a half hours.<br>Content of the Self-Management Program<br>Week 1 Introduction Importance of physical exercise<br>Week 2 Coping with pain, fatigue and stress at work<br>Week 3 Importance of healthy nutrition Problems encountered at work<br>Week 4 Communication techniques at the workplace<br>Week 5 Working with occupational health professionals | Two trainers (one must be a master trainer educated at Stanford University and the other must have received a leaders training by the master trainer) | Participants were recruited through the departments of Human Resource Management from companies and through the practices of general practitioners and occupational health services in the region of Arnhem and Nijmegen in the Netherlands. The inclusion criteria to select participants for the course were: a diagnosed chronic somatic disease like rheumatoid arthritis or diabetes mellitus, having a paid job at the moment of the course, encountering problems at work because of the disease and motivation to follow the course |

| Nr. | **First author (year), country** | **Chronic disease** | **Objectives of intervention** | **Intervention methods** | **Structure of intervention** | **Number and discipline of trainer or counsellors** | **Recruitment procedures** |
|---|---|---|---|---|---|---|---|
| | | | | | Week 6 Plans for the future | | |
| 8 | Friedrich et al. (2005), Austria | Chronic and recurrent low back pain (LBP) | Vocational rehabilitation (VR) program in patients with a rheumatic condition who were at risk for job loss | Exercise Program: The exercise program consisted of individual, submaximal gradually increased exercises. The treatment was directed toward improving spinal mobility, as well as trunk and lower limb muscle length, force, endurance and coordination, thereby restoring normal function<br>Motivational Program + Exercise Program: 1. Extensive counseling and information strategies; 2. Reinforcement techniques were used; 3. The oral agreements between the patient and the therapist were reinforced in writing in the form of a "treatment contract"; 4. Patients were asked to post the treatment contract in a prominent place at home; 5. patients were involved more in their care by reporting all exercises they had performed in an exercise diary. | Exercise program:<br>10 training sessions | 8 physical therapists | Consecutive patients who were referred to the outpatient department of hospital in Vienna, Austria, for individual exercise treatment of LBP were included in the study. Enrollment required LBP fulfilling the topographic criteria, recurrent or chronic LBP of at least 4 months in duration, and patients between 20 and 60 years old. Patients were randomly assigned to 2 groups with regimens<br>that consisted of either the standard exercise program (control<br>group) or a combined exercise and motivational program (motivational<br>group). |
| 9 | Lambeek et al. (2010), Netherlands | CLBP | To evaluate the effectiveness of an integrated care program, combining a patient directed and a workplace directed intervention, for patients with chronic low back pain in comparison with usual care | Aim—To plan and coordinate care and communicate with other involved health care professionals.<br>Workplace intervention: Aim—To achieve consensus of all stakeholders about adjustments to the workplace to facilitate return to work.<br>Graded activity: Aim—To restore patient's occupational function and supervise return to work | Integrated care management by clinical occupational physician: Period—From week 1 to full sustainable return to work, or week 12<br>Content—Formulate treatment plan (week 1), monitor treatment plan (week 6), and, when necessary, communicate with other healthcare professionals<br>Workplace intervention: | Clinical occupational physician, medical specialist, occupational therapist, physiotherapist | The population comprised adults aged 18-65 with low back pain who had visited an outpatient clinic (mainly orthopaedics and neurology, but also rheumatology and neurosurgery) in one of the participating hospitals, had low back pain for more than 12 weeks, were in paid work (paid employment or self-employed) for at least eight hours a week, and were absent or partially absent from work |

| Nr. | **First author (year), country** | **Chronic disease** | **Objectives of intervention** | **Intervention methods** | **Structure of intervention** | **Number and discipline of trainer or counsellors** | **Recruitment procedures** |
|---|---|---|---|---|---|---|---|
| | | | | | Period—From week 3 to week 12<br>Content—Observation of patient's workplace; obstacles on return to work ranked independently by supervisor and patient; patient, supervisor, and occupational therapist brainstorm and discuss possible solutions for obstacles until reaching consensus<br>Graded activity:<br>Period—From week 2 to full sustainable return to work, or after receipt of 26 sessions of graded activity (within maximum of 12 weeks)<br>Content—Baseline (consisting of three sessions) to test patient's functional capacity; individually graded exercise program, teaching patients that, despite pain, moving is safe while increasing activity level | | |

| Nr. | **First author (year), country** | **Chronic disease** | **Objectives of intervention** | **Intervention methods** | **Structure of intervention** | **Number and discipline of trainer or counsellors** | **Recruitment procedures** |
|---|---|---|---|---|---|---|---|
| 10 | Linton et al. (2000), Sweden | Spinal pain | The main focus was to prevent long-term disability by changing patients' behaviors and beliefs so they can cope better with their problems. | *Cognitive-Behavior Therapy Intervention* (CBT). Causes of pain and prevention of chronic problems, managing pain, promoting good health; controlling stress at home and at work, adapting for leisure and work, controlling flare-ups, maintaining and improving results. *Pamphlet Group.* Participants received a previously evaluated pamphlet to read concerning back pain. The pamphlet provided straightforward advice about the best way to cope with back pain by remaining active and thinking positively. *Information Package group*. The information package group received a packet of information once a week for 6 weeks. Each package contained advice and illustrations showing how the patient might cope with spinal pain or prevent it by such methods as lifting properly and maintaining good posture. | CBT: A six-session structured program was offered, in which participants met in groups of 6 to 10 people for 2 hours once a week for 6 weeks. | CBT: Certified behavior therapists | Participants were recruited from local primary care facilities and via an advertisement in a local newspaper. The inclusion criteria required that the participants be 18 to 60 years of age, report less than 3 months of cumulative sick leave during the past year, and have a self-perceived risk of a chronic problem developing. |
| 11 | McGonagle et al. (2014), USA | Ankylosing spondylitis, nerve injury, neuropathy, fibromyalgia, diabetes 1+2, multiple sclerosis), psoriatic arthritis), psychiatric illness and Sjögren's syndrome | Aim—To plan and coordinate care and communicate with other involved health care professionals | The first component is labeled "goal," in which the coach and client agree on the topic(s) for discussion, and the objectives and desired outcomes for the current session. The next component is called "reality", in which the coach helps the client create awareness of their current situation and how it is affecting the identified outcomes or goals. The third component is known as "options," in which possible solutions are identified | six 1-hr phone-based coaching sessions over 12 weeks | Certified coach | Study participants were recruited from two Midwestern U.S. universities, a health insurance provider, and a pharmaceutical organization. 59 participans met enrollment criteria and completed a baseline survey. Enrollment criteria included working an average of at least 30 hr per week, having one or more chronic health conditions that caused difficulties with work, and not planning to retire within 2 years of study enrollment |

| Nr. | First author (year), country | Chronic disease | Objectives of intervention | Intervention methods | Structure of intervention | Number and discipline of trainer or counsellors | Recruitment procedures |
|---|---|---|---|---|---|---|---|
| 12 | Nieuwenhuijsen et al. (2017), Netherlands | Work-related chronic stress complaints | effect of combining mental coaching with light therapy plus pulsed electromagnetic field therapy compared to mental coaching alone | A new treatment platform with light therapy plus Pulsed Electro Magnetic Fields (PEMF) in combination with coaching | IG, PG: 12 weeks, twice a week for 40 min; CG 50 min once a fortnight during a 12-week period | Certified coach, research assistant | Participants had to experience work-related chronic stress complaints and be absent from work for at least 50% of their working contract hours. They had to be diagnosed with neurasthenia (symptoms 0–6 months) and able to speak Dutch. Participants were randomly allocated to one of three groups: group 1 received light therapy/electromagnetic field therapy and coaching (Intervention group); group 2 received the same treatment conditions but the light therapy/electromagnetic field therapy was not activated (Placebo group); and Group 3 received coaching only (Control group). |
| 13 | Ntsiea et al. (2017), South Africa | Stroke | Objective: To determine the effect of a workplace intervention program on the rate of return to work of previously employed stroke survivors | The workplace intervention program was tailored according to functional ability and workplace challenges of each stroke survivor. The control group received usual stroke care which took into consideration job requirements but without workplace intervention | Week 1: Assessment for work skill. Week 2: The therapist interviewed the stroke survivor and employer separately to establish perceived barriers and enablers of RTW. Week 3: A work visit for the stroke survivor to demonstrate what they did at work and identify what they could still do safely and what they could not do. Weeks 4, 5 and 6: continuation of the work visits, while monitoring progress, and making necessary adjustments as per stroke survivor and | Therapist, employer/supervisor | Stroke survivors who were employed at the time of having a stroke were recruited from 2009-2012 from three hospitals which offer stroke rehabilitation services. Included: 1) aged between 18 and 60 years; 2) employed in the formal work sector at the time of stroke; 3) less than eight weeks since onset of stroke (in order to start the workplace intervention program before the end of the six weeks sick leave period) |

| Nr. | First author (year), country | Chronic disease | Objectives of intervention | Intervention methods | Structure of intervention | Number and discipline of trainer or counsellors | Recruitment procedures |
|---|---|---|---|---|---|---|---|
| | | | | | employer's needs (Ntsiea, 2013) | | |
| 14 | Varekamp et al. (2011), Netherlands | Diseases of: musculoskeletal system and connective tissue, nervous system, digestive system, endocrine, nutritional and metabolic diseases, neoplasms, respiratory system, circulatory system | The training program focused on solving work-related problems. A step-wise approach was used: first, workrelated problems were explored and clarified; second, communication at work was addressed; and third, solutions were developed and realized. | Every group session focused on a single theme: (i) exploration and clarification of practical and psychosocial workrelated problems, (ii) insight into feelings and thoughts about having a chronic disease, (iii) communication in daily work situations and role play, (iv) legislation and facilities for disabled employees, (v) communication, assertiveness, and role play, (vi) a SMART (Specific, Measurable, Attainable, Relevant, Time-bound) plan to solve problems, (vii) and a follow-up meeting. | Six 3-hour sessions every two weeks, with a seventh session two months after the sixth. This was combined with three individual counseling sessions | One trainer | Participants were recruited via outpatient clinics, occupational health services, patient organizations and employers |
| 15 | Wong et al. (2008), Hong Kong | mental disorders | The effectiveness and applicability of a supported employment program based on the individual placement and support model | The employment specialist was integrated into the participant's clinical management team, thus facilitating communication between the participant and multidisciplinary professionals. The employment specialist assisted the participant to search for a competitive job on the basis of his or her educational | 20 hours | Employment specialist, clinical management team | Participants were recruited between 2001 and 2003 from patients of the Occupational Therapy Department, Kwai Chung Hospital in Hong Kong. Patients who had been diagnosed at least two years ago as having a mental illness. Inclusion criteria: age between 18 and 55 years (working age range), an interest in competitive employment, not have any serious medical |

| Nr. | First author (year), country | Chronic disease | Objectives of intervention | Intervention methods | Structure of intervention | Number and discipline of trainer or counsellors | Recruitment procedures |
|---|---|---|---|---|---|---|---|
| | | | | background, work preference, and previous work experience. Once employed, on-the-job training and follow-along support was provided to help the individual retain the job for as long a period as possible | | | condition that might affect their longterm ability in competitive work, and willing to participate in the study. |
